# Rational design of GDP‑d‑mannose mannosyl hydrolase for microbial l‑fucose production

**DOI:** 10.1186/s12934-023-02060-y

**Published:** 2023-03-24

**Authors:** Cong Fu, Xuexia Xu, Yukang Xie, Yufei Liu, Min Liu, Ai Chen, Jenny M. Blamey, Jiping Shi, Suwen Zhao, Junsong Sun

**Affiliations:** 1 Shanghai Advanced Research Institute, Chinese Academy of Sciences, Shanghai, 201210 China; 2 School of Life Science and Technology, ShanghaiTech University, Shanghai, 201210 China; 3 University of Chinese Academy of Sciences, Beijing, 100049 China; 4 iHuman Institute, ShanghaiTech University, Shanghai, 201210 China; 5 Fundación Biociencia, José Domingo Cañas, 2280 Ñuñoa, Santiago Chile; 6 Facultad de Química Y Biología, Universidad de Santiago de Chile, 3363 Alameda, Estación Central, Santiago Chile

**Keywords:** l‑Fucose, *Bacillus subtilis*, GDP‑mannose mannosyl hydrolase, GDP‑l‑fucose, Molecular modeling, Microbial production

## Abstract

**Background:**

l‑Fucose is a rare sugar that has beneficial biological activities, and its industrial production is mainly achieved with brown algae through acidic/enzymatic fucoidan hydrolysis and a cumbersome purification process. Fucoidan is synthesized through the condensation of a key substance, guanosine 5′‑diphosphate (GDP)‑l‑fucose. Therefore, a more direct approach for biomanufacturing l‑fucose could be the enzymatic degradation of GDP‑l‑fucose. However, no native enzyme is known to efficiently catalyze this reaction. Therefore, it would be a feasible solution to engineering an enzyme with similar function to hydrolyze GDP‑l‑fucose.

**Results:**

Herein, we constructed a de novo l‑fucose synthetic route in *Bacillus subtilis* by introducing heterologous GDP‑l‑fucose synthesis pathway and engineering GDP‑mannose mannosyl hydrolase (WcaH). WcaH displays a high binding affinity but low catalytic activity for GDP‑l‑fucose, therefore, a substrate simulation‑based structural analysis of the catalytic center was employed for the rational design and mutagenesis of selected positions on WcaH to enhance its GDP‑l‑fucose‑splitting efficiency. Enzyme mutants were evaluated in vivo by inserting them into an artificial metabolic pathway that enabled *B*. *subtilis* to yield l‑fucose. WcaH^R36Y/N38R^ was found to produce 1.6 g/L l‑fucose during shake‑flask growth, which was 67.3% higher than that achieved by wild‑type WcaH. The accumulated l‑fucose concentration in a 5 L bioreactor reached 6.4 g/L.

**Conclusions:**

In this study, we established a novel microbial engineering platform for the fermentation production of l‑fucose. Additionally, we found an efficient GDP‑mannose mannosyl hydrolase mutant for L‑fucose biosynthesis that directly hydrolyzes GDP‑l‑fucose. The engineered strain system established in this study is expected to provide new solutions for l‑fucose or its high value‑added derivatives production.

**Supplementary Information:**

The online version contains supplementary material available at 10.1186/s12934-023-02060-y.

## Introduction

L‑Fucose (6‑deoxy‑l‑galactose) is an indispensable component of mammalian glycans and glycolipids, and the O‑fucosylation of glycans and glycolipids is biologically important due to its effect on protein function [1]. l‑Fucose also exhibits unique functional properties with regard to metabolic regulation, immunological responses and pathogen resistance. It has been reported that l‑fucose decreased fat accumulation and hepatic triglycerides in mice fed a high‑fat diet, helping to restore healthy intestinal composition and function [2]. l‑Fucose is known to suppress pathogen virulence, protecting the host against infection and inflammation [3], and l‑fucose‑containing glycan motifs alter pro‑ and anti‑inflammatory immunoglobulin G activities, which indicates their potential application in immunotherapy [4]. Countless studies on the physiological mechanisms of l‑fucose have led to its application in the treatment of human diseases. Research has found that ∼20% of the population has a Fut2 defect, which is linked to diseases with potential connections to the gut microbiota [5, 6], while the presence of l‑fucose enhances fitness by supporting beneficial gut bacteria [7]. Interestingly, l‑fucose is also a common constituent of extracellular polysaccharide (EPS) secreted by many gram‑negative pathogens [8]. Oral l‑fucose supplementation has been reported as a promising therapy for congenital glycosylation disorders, improving growth and cognitive skills in children with GFUS‑CDG (GDP‑l‑fucose synthase‑Congenital disorders of glycosylation) [9]. l‑Fucose can also be applied as the substrate for producing 2′‑fucosyllactose (2′‑FL) and 3‑fucosyllactose (3‑FL), two key oligosaccharides in human milk [10, 11]. A recent study reported the chemical synthesis of l‑fucose‑containing colanic acid hexasaccharide [12], and it is believed that other valuable fucosylated glycans can be synthesized from l‑fucose.l‑Fucose exists in the form of sulfated l‑fucose‑rich polysaccharides called fucoidans in brown algae, seaweeds and echinoderms [13]. Industrial‑level l‑fucose production is achieved by laborious and costly extraction and purification processes using brown algae as the crude material [14]. Other routes for fabricating l‑fucose have been developed to meet the increasing demand for this chemical. For example, l‑fucose can be chemically converted from d‑mannose [15], and it can also be extracted from the enzymatic hydrolysate of l‑fucose‐rich microbial EPS [16] or bio converted from rarer monosaccharides, such as l‑fuculose [17].

**Fig. 1:**
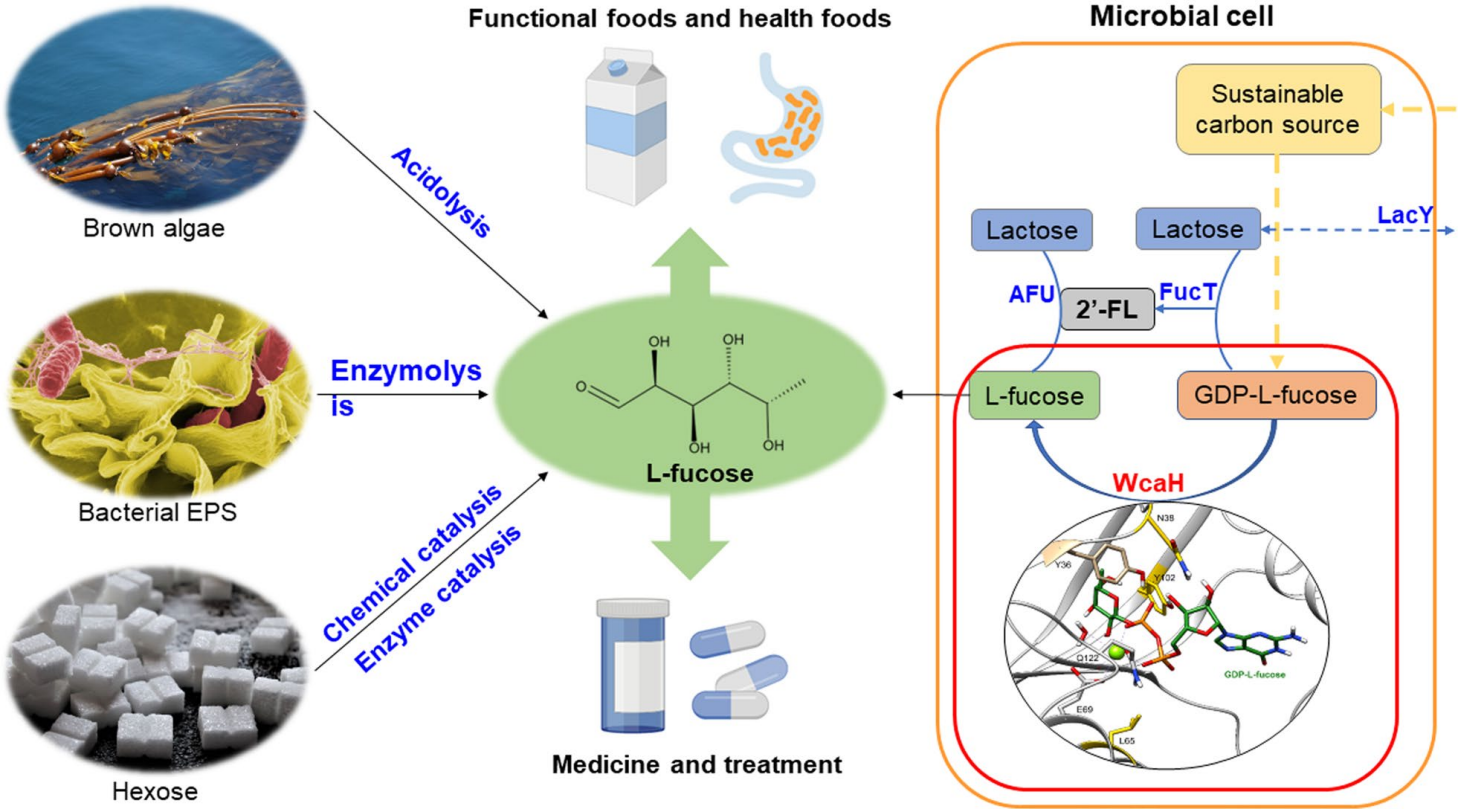
Schematic for L‑fucose production and application. Previously reported L‑fucose production methods are shown in blue font, and the synthesis method reported in this work is shown in red font. LacY: lactose permease; FucT: 2′‐fucosyltransferase; AFU: α‐L‐fucosidase; WcaH: guanosine 5’‑diphosphate (GDP)‑mannose mannosyl hydrolase

Synthetic biology has greatly expanded our ability to competitively produce high‑value chemicals through the rational design of metabolic networks and microbial strain engineering [18, 19]. Pipeline design and construction for l‑fucose bioproduction from sustainable carbon sources seems to be a more promising approach. Previously engineered *Escherichia coli* was developed to produce l‑fucose from in situ synthesized 2′‐fucosyllactose (2′‐FL) by recombinant 2′‐fucosyltransferase and α‐l‑fucosidase [20]. As demonstrated in Fig. 1, such a microbial l‑fucose synthetic route, involving the addition and discharge of a lactose molecule, would be redundant if guanosine 5′‑diphosphate (GDP)‑l‑fucose could be directly degraded into GDP and L‑fucose; however, no enzyme has been identified as being capable of such a catalytic reaction. A known enzyme with a similar function is *E. coli* GDP‑mannose mannosyl hydrolase (GDPMH, also known as WcaH). *wcaH* is part of an operon encoding enzymes for the synthesis of colanic acid, a l‑fucose‑rich extracellular polysaccharide. WcaH is speculated to regulate the cellular level of GDP‑d‑mannose by catalyzing the hydrolysis of GDP‑D‑mannose to GDP and d‑mannose in the presence of Mg^2+^ to limit the carbon flux channeled toward GDP‑l‑fucose formation [21]. Interestingly, the acceptable substrates of WcaH are GDP‑d‑mannose, GDP‑d‑glucose and GDP‑l‑fucose, all of which bind to the catalytic center of the enzyme through 12 hydrogen bonds [22], although for GDP‑l‑fucose, the *k*_*cat*_ of WcaH is low, with a high *K*_*m*_, suggesting that GDP‑l‑fucose might be a potent competitive inhibitor of GDP‑d‑mannose hydrolysis [23]. Thus, WcaH is potentially a good candidate for the direct hydrolysis of GDP‑l‑fucose to yield l‑fucose (Fig. 1).

Regarding the main applications of l‑fucose in the food or pharmaceutical fields, among the common bacterial hosts, *Bacillus subtilis* is favored over *E. coli* since the latter produces lipopolysaccharides (LPS) that can trigger aberrant inflammation. *B*. *subtilis* is regarded as generally recognized as safe (GRAS) and has become a model chassis host cell for industrial applications due to its robustness and metabolic versatility [24]. A variety of valuable compounds, including small molecular compounds, proteins and biopolymers, have been produced with engineered *B*. *subtilis* microbial platforms [25–27]. However, *B. subtilis* lacks the GDP‑l‑fucose generation pathway that *E. coli* naturally possesses. To achieve enzymatic l‑fucose production from GDP‑l‑fucose, an *E. coli*‑derived GDP‑l‑fucose generation pathway has to be transferred into *B. subtilis* ATCC 6051a, and similar work has been accomplished for microbial 2’‑FL production [28]. To produce l‑fucose with engineered *B*. *subtilis*, this work began with rationally designing WcaH mutants based on the docking model of wild‑type WcaH, followed by expressing the GDP‑l‑fucose synthetic pathway together with WcaH or its mutants. This is the first report of microbial l‑fucose production through GDP‑l‑fucose hydrolysis.

## Results and discussion

### Construction of a de novo pathway for l‑fucose synthesis in*** B. subtilis***

The host *B*. *subtilis* 6051a (namely, *B*. *subtilis* 164) used in this study is a well‑studied chassis strain with a clear genetic background and is easy to cultivate at a high density [29]. In addition, *B*. *subtilis* is generally recognized as safe (GRAS) by the FDA and is suitable for the production of food processing enzymes or food additives [26, 30–32]. The pyrogen‑free nature of *B*. *subtilis* also offers advantages in product separation and purification. To assess whether *B*. *subtilis* was able to accumulate l‑fucose during cultivation, a *B*. *subtilis* growth experiment was performed. As shown in Additional file 1: Fig. S1, no colony was formed when cells were plated on solid medium containing l‑fucose as the sole carbon source, indicating that *B*. *subtilis* 164 lacks the capability to catabolize l‑fucose. Next, to facilitate the convenient genetic manipulation of *B*. *subtilis* 164, we improved the transformation efficiency by integrating *comk* (*B*. *subtilis* endogenous gene for the regulation of genetic competence and DNA uptake) into the genome of host cells under the control of a mannitol‑inducible P_*mtlA*_ promoter, and the generated strain was designated 164 M. However, there are no efficient expression kits for *B*. *subtilis* 164 M. To facilitate the efficient expression of target pathway, we attempted to transplant a T7 expression system into the 164 M genome at the *aprE* locus by integrating a DNA cassette expressing T7 RNA polymerase (T7 RNAP), which generated 164MT.

In some gram‑negative intestinal bacteria, GDP‑l‑fucose is used as the l‑fucose donor to synthesize extracellular polysaccharides or lipopolysaccharides. For example, colanic acid (M‑antigen EPS) produced by *E*. *coli* has a specialized operon for expressing the enzymes required to perform glycosyl‑transferring reactions and generate GDP‑l‑fucose from d‑mannose, including WcaH. However, l‑fucose accumulation has not been reported, probably due to the low catalytic efficiency of WcaH for GDP‑l‑fucose. In addition, endogenous *fucI*, *rhaA*, and *araA* may also metabolize trace l‑fucose even if it is accidentally produced. Nevertheless, the *E*. *coli* pathway that enables GDP‑l‑fucose production together with WcaH or its mutants was transferred into *B*. *subtilis.* In contrast with *E. coli*, there is no known l‑fucose metabolizing pathway in *B. subtili*s, which would be a major advantage for l‑fucose accumulation.

**Fig. 2:**
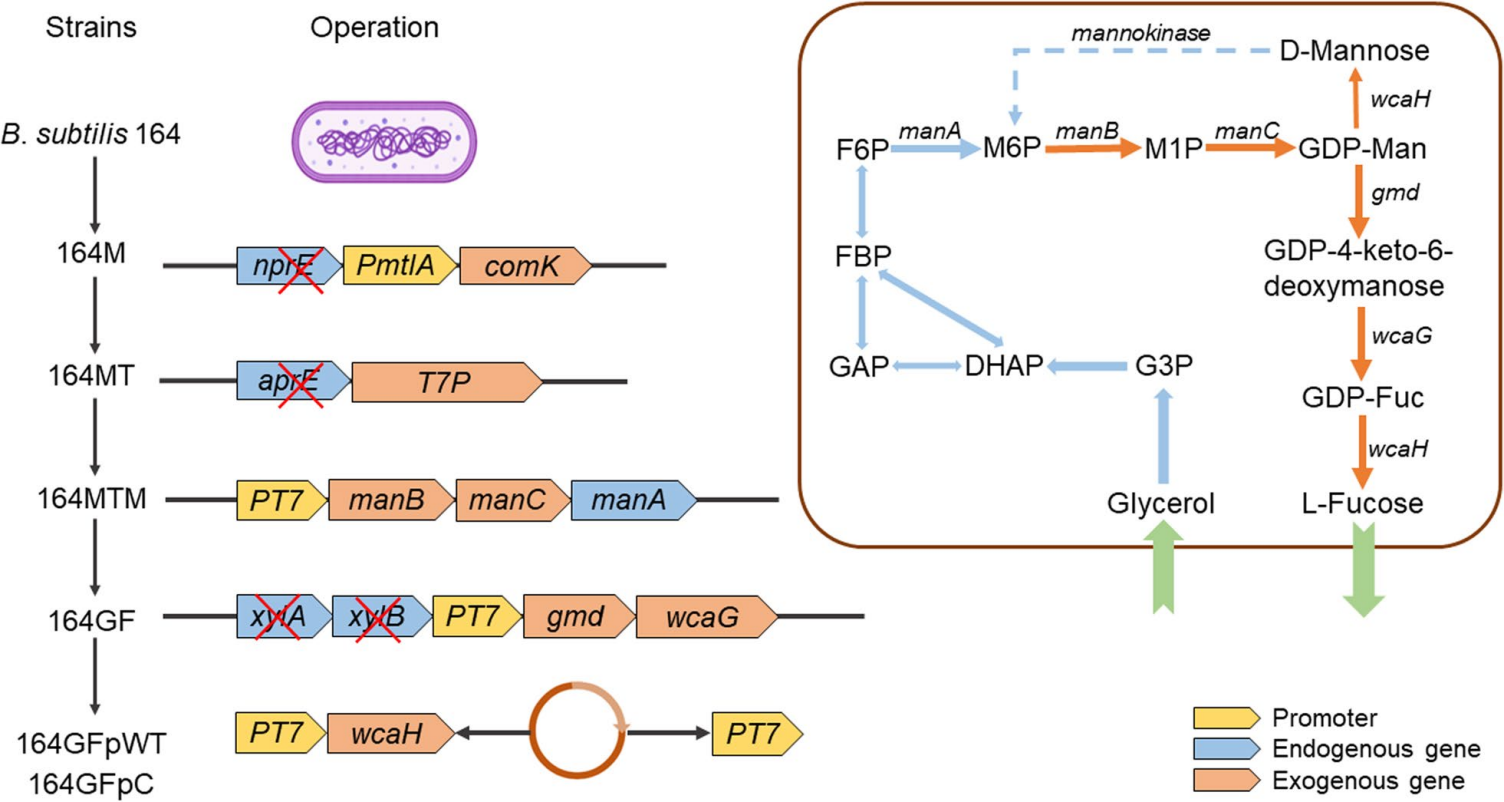
Schematic diagram of the main metabolic pathways involving L‑fucose generation and the genotypes of *B. subtilis* strains leading to 164GFpWT, a strain capable of L‑fucose production after incorporating a vector expressing WcaH. G3P: glycerol‑3‑phosphate; DHAP: dihydroxy acetone‑phosphate; GAP: glyceraldehyde‑3‑phosphate; FBP: fructose‑1,6‑diphosphate; F6P: fructose‑6‑phosphate; M6P: mannose‑6‑phosphate; M1P: mannose‑1‑phosphate; GDP‑Man: GDP‑D‑mannose; GDP‑Fuc: GDP‑L‑fucose

As shown in Fig. 2, the GDP‑l‑fucose formation pathway was cumulatively expressed by two artificial operons that were then engineered into 164MT. The first operon consisted of *manA*, *manB* and *manC*. *manA* encodes a native mannose‑6‑phosphate (M6P) isomerase that transforms fructose‑6‑phosphate (F6P) to M6P, which is then used as the substrate of phosphomannomutase (ManB) and catalyzed to mannose‑1‑phosphate (M1P). M1P is further converted into GDP‑D‑mannose (GDP‑Man) through the catalysis of mannose‑1‑phosphate guanyltransferase (ManC). The second operon contained coding sequences of GDP‑mannose 4, 6‑dehydratase (Gmd) and GDP‑fucose synthetase (WcaG). Gmd and WcaG convert GDP‑Man to GDP‑L‑fucose. Both operons were cloned behind the T7 promoter, and *manA*/*manB*/*manC* cassette was inserted at the *manA* locus in 164MT, resulting in 164MTM. Next, *gmd*/*wcaG* cassette was integrated at the *xylA* locus in the 164MTM genome, leading to 164GF, which was then transformed by the WcaH‑expressing plasmid pMK4‑T7wcaH. The transformant bearing pMK4‑T7wcaH was designated 164GFpWT. As the control, pMK4‑T7 was transformed into 164GF, generating 164GFpC (Fig. 2).

**Fig. 3:**
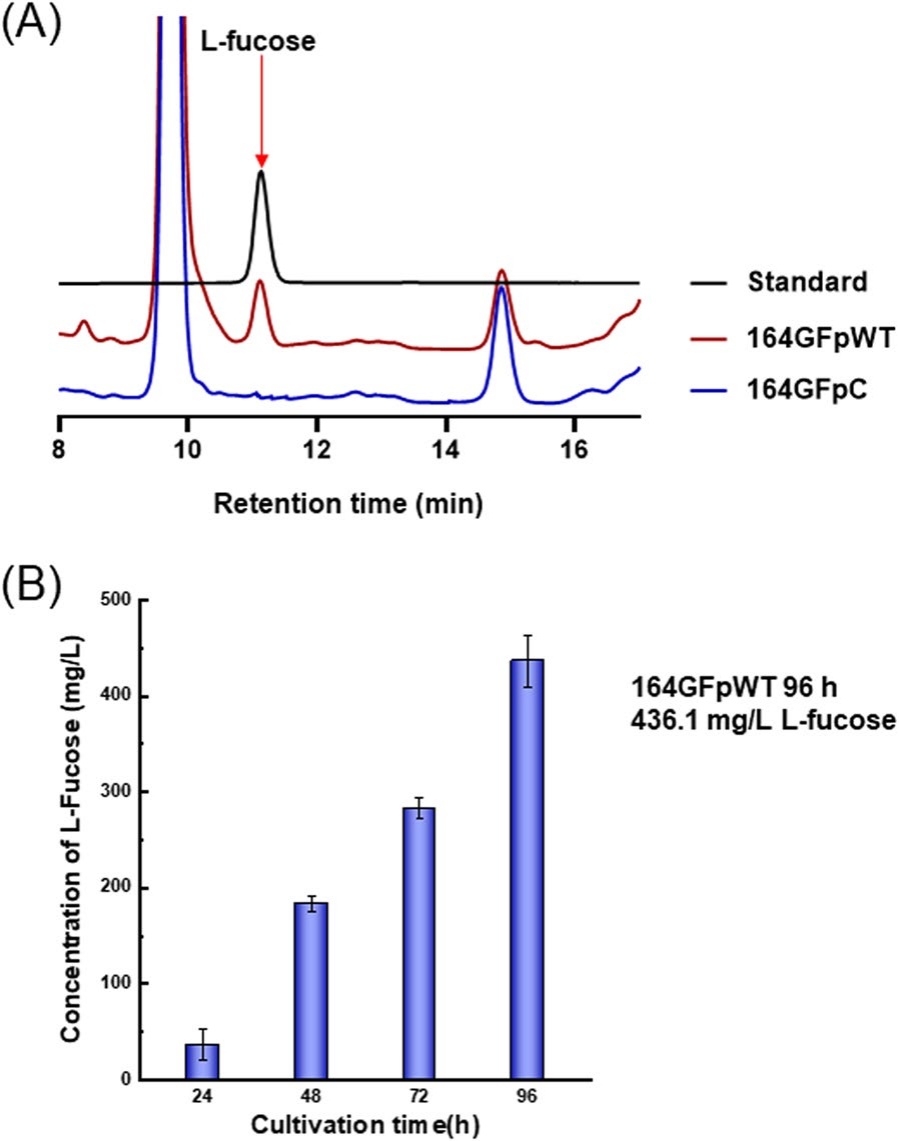
Detection of L‑fucose by high‑performance liquid chromatography (HPLC). **A** HPLC profiles of cell‑free broths from the control strain and the WcaH‑expressing strain. **B** L‑fucose accumulated during 164GFpWT fermentation, as detected by HPLC

Both 164GFpC and 164GFpWT were cultivated in shake flasks containing Luria‑Bertani (LB) medium for 96 h, with glycerol supplemented as an extra carbon source. The cell‑free broth was collected as a sample for high‑performance liquid chromatography (HPLC) analysis during growth. As shown in Fig. 3A, a compound with the same retention time as l‑fucose was found in the culture broth of 164GFpWT but not in the culture broth of 164GFpC. The compound was further identified to be l‑fucose following liquid chromatography‑mass spectrometry (LC‑MS) analysis, with m/z values of 164.10 (l‑fucose), 182.16 (l‑fucose·H_2_O), and 147.08 (l‑fucose‑OH^‑^), corresponding to the molecular ion of l‑fucose (Additional file 1: Fig. S2). The l‑fucose accumulation in the broth of 164GFpWT reached 436.1 ± 27.5 mg/L after 96 h of cultivation (Fig. 3B). The cellular lysate of the cultures was also sampled and analyzed by HPLC, and almost no L‑fucose was found, indicating that nearly all L‑fucose was excreted out of the cells. WcaH can also break the native substrate GDP‑d‑mannose to yield d‑mannose, but d‑mannose was not detected in the fermentation broth by HPLC analysis. This fact is supposed to be caused by two reasons, first, *B. subtilis* can metabolize d‑mannose through mannokinase (or hexokinase) and enter glycolysis pathway; second, l‑fucose produced competitively inhibit the native catalytic reaction that should have yield d‑mannose. Herein, a de novo l‑fucose synthetic pathway was successfully constructed in a *B*. *subtilis* strain that was capable of l‑fucose production during growth. In contrast to the reported l‑fucose‑producing *E. coli* system [22], the engineered *B. subtilis* bypassed 2′‑FL formation and breakage. It is interesting that there has never been any report demonstrating that *E. coli* was able to synthesize l‑fucose utilizing its native GDP‑l‑fucose‑forming pathway and wild‑type (WT) WcaH, indicating that in addition to the l‑fucose metabolizing enzymes, sophisticated regulation mechanisms may also exist to guide GDP‑l‑fucose only toward polysaccharide synthesis, suggesting that the delicate engineering of *E. coli* native pathways is another feasible way to produce l‑fucose.

### Modeling‑based mutation of the l‑Fucose C6 methyl binding sites of WcaH

**Fig. 4:**
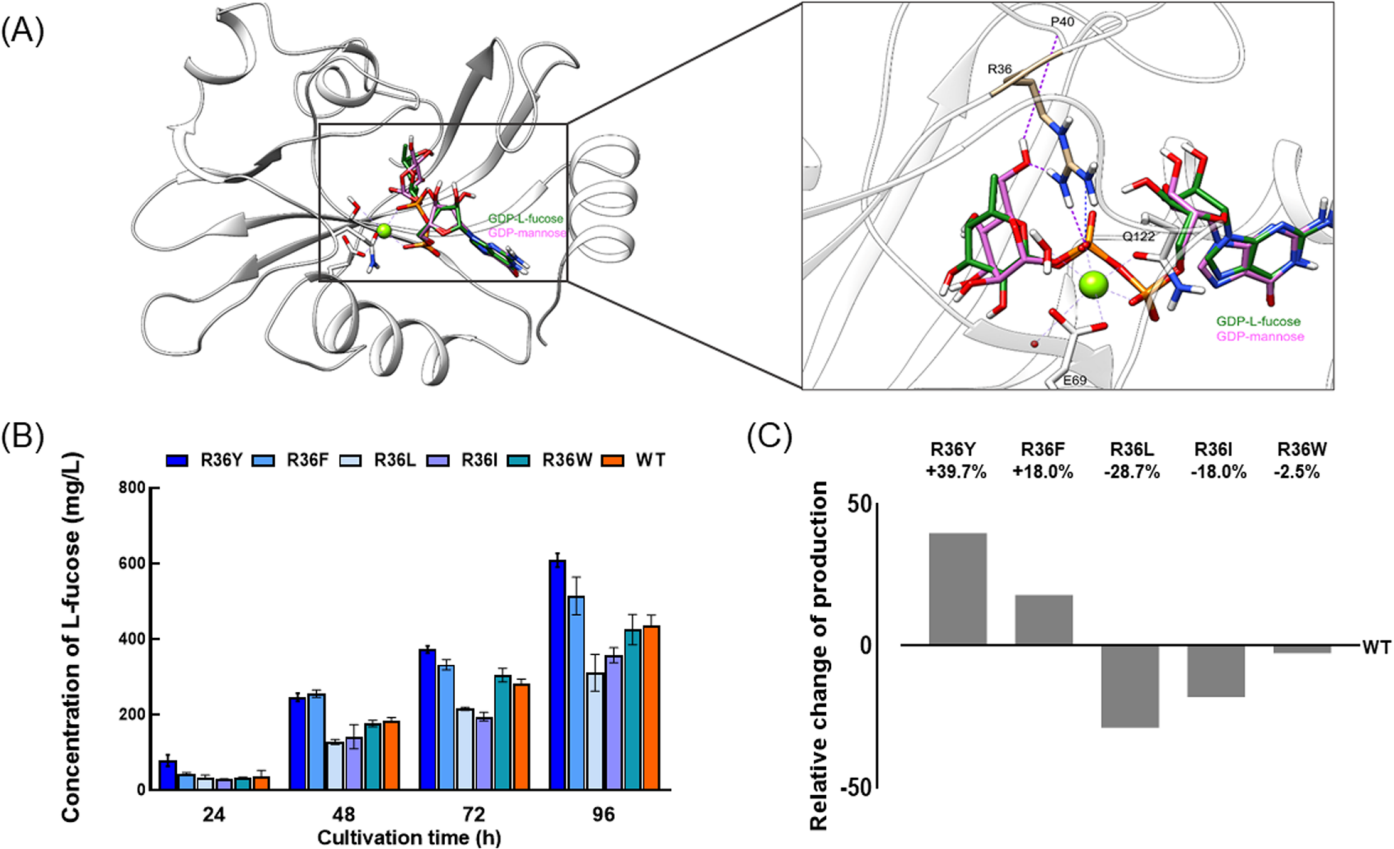
Modeling of WcaH, the R36 site mutations and the effects on L‑fucose production by *B. subtilis* strains. **A** Structure of WcaH binding with GDP‑L‑fucose (green) and GDP‑D‑mannose (pink) and residue R36. **B** L‑fucose accumulated in the broths of different strains. **C** Comparison of L‑fucose produced by strains expressing wild‑type WcaH‑ or R36‑modified enzymes

WcaH is a type of MutT nucleoside triphosphate pyrophosphohydrolase belonging to a protein family of Nudix hydrolases. WcaH binds with Mg^2+^/Mn^2+^ and the triphosphate moiety of the substrate in a novel loop‑helix‑loop motif that is conserved in the MutT family of proteins, and its homology modeling has been established by using published X‑ray structures as templates [33]. The chemical difference between GDP‑L‑fucose and GDP‑d‑mannose is the inclusion of a methyl group on the C6 of L‑fucose instead of a hydroxymethyl group in d-mannose (Fig. 4A). Structural analysis of the complex of WcaH combined with GDP‑d‑mannose or GPD‑l‑fucose revealed that the R36 of WcaH is responsible for binding the hydroxyl group on C6 of GDP‑d‑mannose. In addition, there is a hydrogen bond between R36 and the C6 hydroxyl, and R36 can form a salt bridge with the second phosphate radical on GDP, which helps to stabilize the intermediate state formed during catalysis. Moreover, GDP‑l‑fucose is not able to form an efficient connection with R36, which may account for the low catalytic activity of WcaH for GDP‑l‑fucose. To potentially enhance the GDP‑l‑fucose‑hydrolyzing activity of WcaH, R36 can be replaced by a hydrophobic amino acid, which supposedly stabilizes the methyl group on the C6 of l‑fucose. For that purpose, R36 was substituted for aromatic amino acids, such as R36Y, R36F, and R36W. Another strategy is to form a stabler salt bridge between the enzyme and GDP‑l‑fucose in the intermediate state by changing R36 to an aliphatic amino acid, such as isoleucine (R36I) or leucine (R36L), which might provide a more hydrophobic microenvironment for the methyl group on the C6 of l‑fucose.

Thus, 164GF strain harboring vectors expressing WcaH^R36Y^, WcaH^R36F^, WcaH^R36L^, WcaH^R36I^, and WcaH^R36W^ were constructed and tested for L‑fucose production with growth experiments. As shown in Fig. 4B, l-fucose accumulated gradually and reached its maximum level after 96 h of fermentation. The highest titers of l-fucose during 96 h cultivation by WcaH^R36Y^, WcaH^R36F^, WcaH^R36L^, WcaH^R36I^, and WcaH^R36W^ reached 609.3 mg/L, 514.5 mg/L, 310.7 mg/L, 357.7 mg/L, and 425.3 mg/L, respectively, compared to 436.1 mg/L achieved by WT WcaH. Thus, 39.7% and 18.0% increases in l-fucose were achieved by WcaH^R36Y^ and WcaH^R36F^, respectively (Fig. 4C). Surprisingly, the GDP‑l-fucose‑hydrolyzing abilities of WcaH^R36L^ and WcaH^R36I^ seemed to be dramatically lowered.

### Enzyme design around the phosphate radical of GDP

**Fig. 5:**
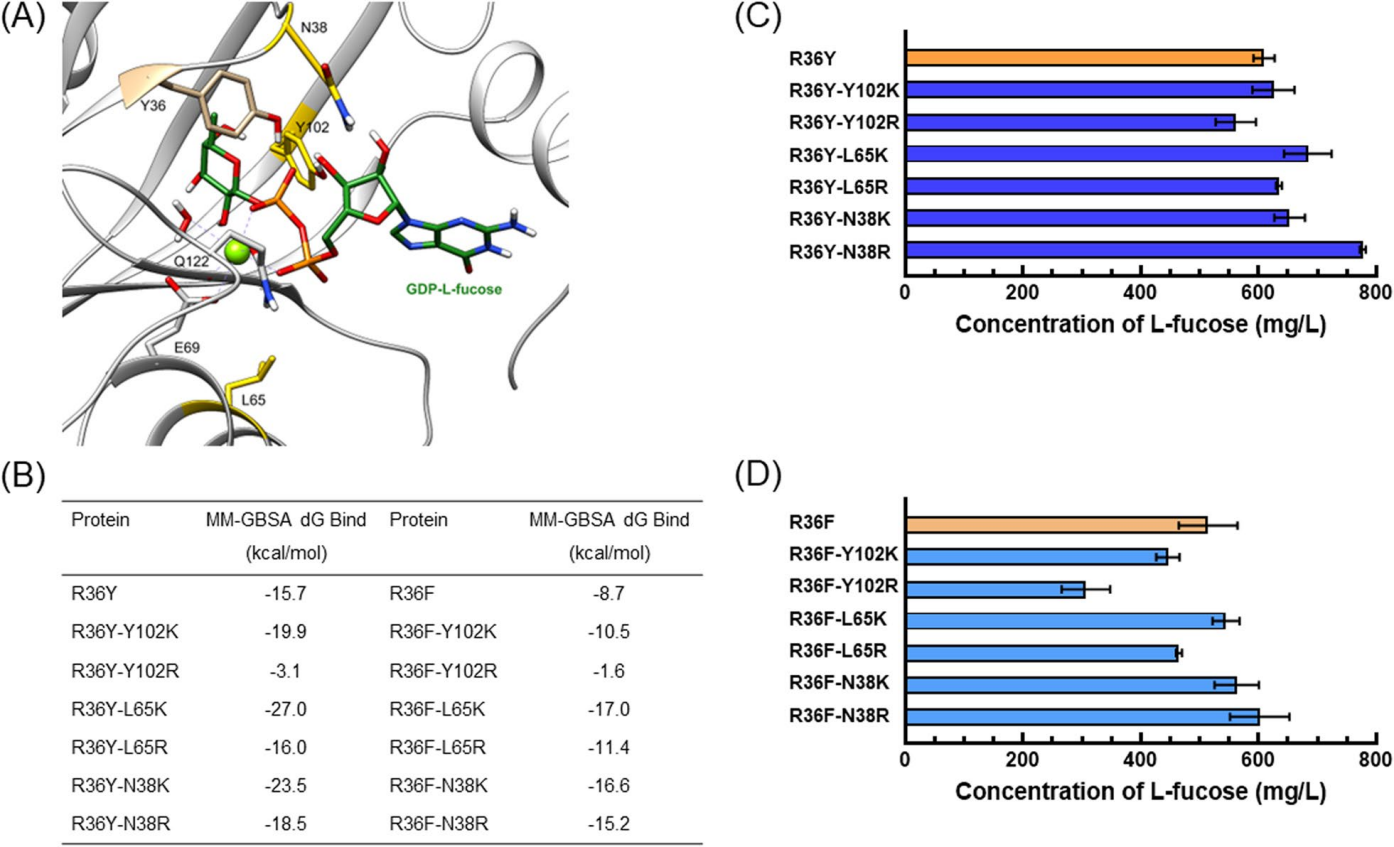
Mutation of the site around the phosphate radical of GDP‑L‑fucose. **A** Structure of the WcaH active center around the phosphate groups. **B** MM/GBSA‑based free binding energy prediction of the enzymes upon binding GDP‑L‑fucose. **C** L‑fucose production of WcaH^R36Y^ and its derived mutants. **D** L‑fucose production of WcaH^R36F^ and its derived mutants

Following the investigation of the interaction between GDP‑l-fucose and the WcaH catalytic center, amino acid residues enclosing the phosphate radical were further projected and rationally designed based on WcaH^R36Y^ and WcaH^R36F^. We speculated that the changes occurred on the binding sites on phosphate moiety may change the position or the orientation of the substrate in the pocket, so that it may lead to improved catalytic efficiency. Then N38R/K, L65R/K and F102R/K were designed and deduced to be able to stabilize the intermediate state of the enzyme/substrate. We speculated that those alkaline amino acids could form salt bridges or hydrogen bonds with the phosphate radical of GDP. Among them, L65R/K might act on the first phosphate radical in GDP, while N38R/K and F102R/K might stabilize the second phosphate radical. The molecular mechanics‑generalized born surface area (MM‑GBSA) method was used to calculate the binding free energy to evaluate the thermodynamic stability. As shown in Fig. 5B, most of the mutant enzymes were more stable than WT WcaH with respect to the binding of GDP‑l-fucose, except for Y102R performed on R36Y or R36F (Fig. 5B); therefore, most of those mutants exhibited higher catalytic activity for the substrate.

Based on the modeling analysis, N38R/K, L65R/K, or F102R/K was introduced into WcaH^R36Y^ or WcaH^R36F^, and the l-fucose production potential of the resultant mutants was evaluated thereafter through growth experiments. As shown in Fig. 5C and D, the l-fucose titers after 96 h of cultivating WcaH^R36Y/F102K^, WcaH^R36Y/F102R^, WcaH^R36Y/L65K^, WcaH^R36Y/L65R^, WcaH^R36Y/N38K^, and WcaH^R36Y/N38R^ reached 625.6 mg/L, 561.5 mg/L, 683.8 mg/L, 634.8 mg/L, 652.9 mg/L, and 777.3 mg/L, respectively. Moreover, the L‑fucose titers of WcaH^R36F/F102K^, WcaH^R36F/F102R^, WcaH^R36F/L65K^, WcaH^R36F/L65R^, WcaH^R36F/N38K^, and WcaH^R36F/N38R^ were determined to be 445.9 mg/L, 306.6 mg/L, 544.8 mg/L, 464.8 mg/L, 562.9 mg/L, and 602.1 mg/L, respectively. Notably, the double mutants derived from WcaH^R36Y^ had comparatively higher l-fucose titers than their counterparts derived from WcaH^R36F^, which is consistent with the results observed from the related single mutations. Among all of the mutants, WcaH^R36Y/N38R^ demonstrated the best l-fucose production performance, with a 27.6% increase compared to that of the single mutant WcaH^R36Y^.

Modeling and simulating the structures of enzyme‑substrate complexes has become a common method in enzyme engineering, and structure‑guided design at the active site can not only enhance the catalytic capacity of enzymes but also facilitate gain‑of‑function for the synthesis of new products in many studies [34–36]. In this manner, we have successfully developed a “better” GDP‑l-fucose‑hydrolyzing enzyme by rational design and the site‑directed mutagenesis of WT WcaH. The amino acid residue at position 36 was confirmed to be a key residue for substrate binding. The nonpolar amino acid side chain generated by mutation at position 36 may form a suitable fucosyl‑binding pocket for GDP‑l-fucose. Amino acid residues around the phosphate group of GDP‑l-fucose were also found to affect the activity of WcaH. Consequently, when the asparagine residue at position 38 was changed into a basic amino acid arginine residue, the activity of engineered WcaH for GDP‑l-fucose was further increased. In addition to the rational design of key binding sites reported in this work, more mutagenesis libraries involving other structural sites should be considered and investigated to further increase the enzymatic efficiency.

### Systematic improvement of the cellular platform to improve l‑Fucose production

**Fig. 6:**
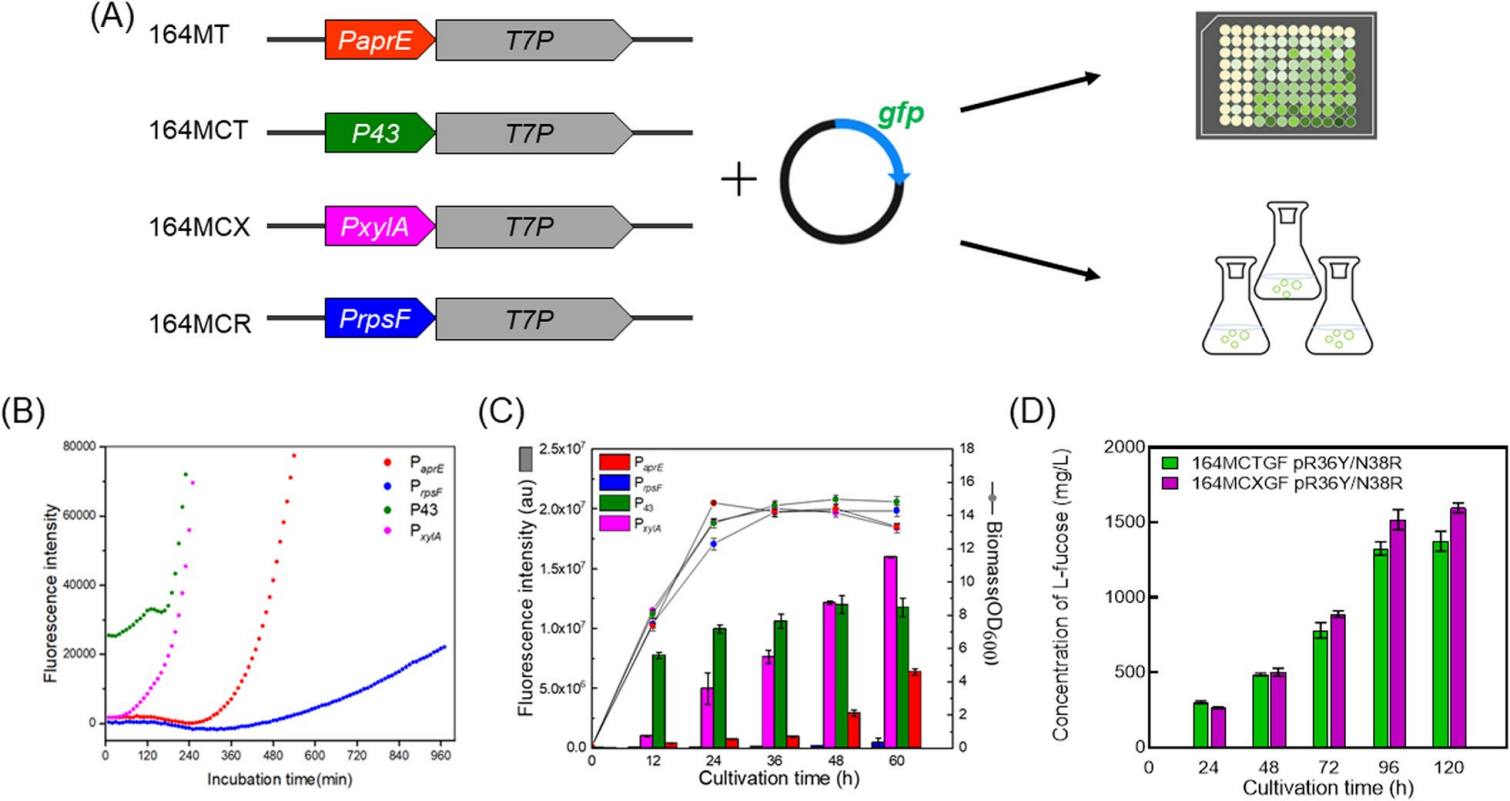
Evaluation of the expression strength of T7 RNA polymerase on the level of GFP transcribed under a T7 promoter. **A** Schematic diagram of the evaluation system, including the placement of different promoters for the transcription of T7 RNAP and the construction and assay of GFP‑expressing strains. **B** Automatic monitoring of the fluorescence intensity of cultures incubated in 96‑well microplates. **C** Fluorescence intensity of strains cultivated in shaking flasks. **D** L‑fucose production of strains equipped with T7 RNAP driven by P_43_ (164MCTGF) or P_*xylA*_ (164MCXGF)

Recombinant T7 RNAP is one of most important biological applications [37], and the construction of a T7 RNAP/T7 promoter‑based heterologous expression system in *B. subtilis* is vital for l-fucose production. To investigate the impact of the level of T7 RNAP on heterologous peptides as well as the l-fucose synthesis efficiency, different promoters that drive T7 RNAP expression were evaluated systematically. To achieve this, the coding DNA fragment of T7P was integrated at the *aprE* locus under P_*aprE*_, the native *aprE* promoter, and three other promoters, including the constitutive promoter P_43_, P_*xylA*_, a xylose‑inducible promoter, and P_*rpsF*_, that specifically and efficiently remained active during the logarithmic phase [38], producing strains that included 164MT, 164MCT, 164MCX, and 164MCR, respectively (Fig. 6A). Next, plasmid pMK4‑T7‑gfp was constructed and transformed into those strains. To assess the promoter strength, GFP‑expressing strains were cultivated in 96‑well microplates or in 250 ml shaking flasks containing liquid LB medium. Green fluorescent protein (GFP) fluorescence was automatically monitored by a microplate reader between 0–960 min (Fig. 6B) or manually measured using a spectrometer between 0–60 h (Fig. 6C).

As shown in Fig. 6B, during early logarithmic growth (0–120 min), P_43_ and P_*xylA*_ led to the other two promoters in the expression of GFP. During shake‑flask growth (Fig. 6C), P_43_ remained active after 12 h during the whole cultivation period; however, its accumulated GFP fluorescence was surpassed by that of P_*xylA*_ after 48 h, whereas P_*rpsF*_ exhibited the lowest promoter efficiency. Based on these results, we speculated that T7 RNAP is better transcribed by P_*xylA*_ or P43 when all of the components of the artificial synthesis pathway are placed under the T7 promoter. Therefore, growth experiments were performed using the engineered *B. subtilis* strains 164MCTGF and 164MCXGF that harboring pMK4-R36Y/N38R, and the accumulated l-fucose titers were monitored during growth. As seen in Fig. 6D, after 120 h of cultivation, 164MCXGF produced 1600.4 mg/L of L‑fucose, which was 16.4% higher than that produced by 164MCTGF (1375.4 mg/L).

### Fed‑batch fermentation for l‑Fucose production

**Fig. 7:**
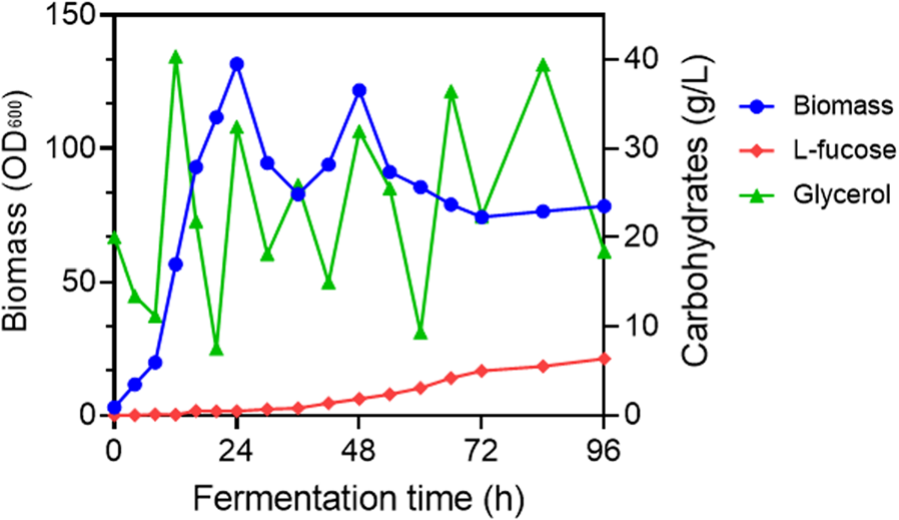
Concentration profiles of L‑fucose, glycerol and biomass during the fed‑batch growth of 164MCTGF

To evaluate the feasibility of l-fucose scale‐up production, fed‑batch fermentation was further performed. Respectively, 164MCTGF pR36Y/N38R and 164MCXGF pR36Y/N38R were fermented in bioreactors in preliminary study, and their productivity was quite close. For the need of inducer‑free during growth, we use 164MCTGF pR36Y/N38R for further optimizing fermentation study. 164MCTGF pR36Y/N38R was cultivated for 96 h in 5 L bioreactors, each containing 2.5 L of LB supplemented with 800 g/L glycerol as the feeding source. As shown in Fig. 7, the l-fucose yield increased progressively, with the highest L‑fucose titer reaching 6.4 g/L within 96 h, which was 4 times that achieved during shake‑flask growth. The highest cell density was achieved at 24 h, with an OD_600_ value of 131.7. Glycerol was replenished into the bioreactor during fermentation and maintained at more than 10 g/L, and 18.4 g/L glycerol was left unconsumed in the broth at 96 h. The strain produced 6.4 g/L l-fucose within 96 h, reaching a productivity of 0.06 g/L/h during that period.

Therefore, by introducing rationally designed WcaH into an engineered *B. subtilis* strain, for the first time, we created a new fermentation biotechnology for l-fucose production that was independent of the chemical or enzymatic lysis of fucoidan or fucosyllated oligosaccharides. While fucosylation is considered to be an important modification that endows molecules with diverse structures and biological activities, L‑fucose itself can be used for pharmaceutical, medical nutrition and cosmetic applications. This novel bioproduction route paves the foundation for large‑scale l-fucose fermentation production and commercialization in the future.

## Conclusion

In summary, we engineered *B*. *subtilis* for the production of a valuable monosaccharide, l-fucose, from an economic carbon source combined with the rational design of GDP‑d‑mannose mannosyl hydrolase and de novo construction of the related pathway. This work is the first to report l-fucose biosynthesis via an artificial pathway that directly hydrolyzes GDP‑L‑fucose. The expression system and pathway constructed in this study not only provide a sustainable method for food‑grade l-fucose production but also provide a platform for the synthesis of other valuable sugars derived from l-fucose.

## Materials and methods

### Strains, plasmids and medium

The strains and plasmids used in this study are listed in Additional file 1: Table S1. The primers used in this study are listed in Additional file 1: Table S2. *B*. *subtilis* ATCC 6051a was purchased from the American Type Culture Collection (ATCC) and used for genetic manipulation in this study. *E*. *coli* DH5α was used for vector construction, propagation and preservation, and its genomic DNA was applied as the template for the polymerase chain reaction (PCR) preparation of DNA fragments derived from *E*. *coli*. pMK4, a gift from the Bacillus Genetic Stock Center (BGSC), was used as the backbone to construct *B. subtilis* expression vectors. For recombinant expression, *wcaH* (NCBI Gene ID: 946,559) was cloned behind a T7 promoter on pMK4‑T7.

LB medium (yeast extract 5 g/L, tryptone 10 g/L, NaCl 10 g/L) was used for the cultivation of *E*. *coli* and *B*. *subtilis*. Routine growth experiments were performed at 37 ℃ with shaking rotors at 200 rpm, and chloramphenicol (10 mg/L), ampicillin (100 mg/L) and erythromycin (10 mg/L) were added to the medium as needed.

### Growth experiments

For shake‑flask growth, a *B*. *subtilis* single colony was inoculated into 5 mL of liquid LB and incubated at 37 ℃ overnight with shaking at 200 rpm. Then, 1 mL of grown culture was transferred to 50 mL of modified LB (LB with extra glycerol, 20 g/L) in a 250/500 mL shake flask and grown at 37 ℃ with shaking at 200 rpm. Samples were collected at regular time intervals for HPLC and LC–MS analyses. For fed‑batch fermentation, an overnight culture of 50 mL LB grown in a 500 mL shake flask was transferred to a 5 L bioreactor with 3.0 L of fermentation medium that contained 20 g/L glycerol, 5 g/L yeast extract, 20 g/L tryptone, 2 g/L MgSO_4_·6H_2_O_,_ 10 g/L (NH_4_)_2_HPO_4_, and 5 g/L KCl. The medium pH was maintained at 6.5 using aqueous ammonia as an alkali supplement and 500 g/L glycerol as a carbon source supplement. The growth temperature was set at 37 °C, with the ventilation volume set at 3.0 vvm and an initial stirring speed of 500 rpm.

### Genetic manipulations

Vector DNA construction was mostly accomplished by using the ClonExpress II One Step Cloning Kit (Vazyme Biotech Co., Ltd.). Linear DNA fragments for homologous recombination were constructed via the overlapping PCR technique. For instance, the P_*mtlA*_‑comk cassette contained a copy of *comk* assembled behind the *mtlA* promoter, homologous arms (UnprE and DnprE) for recombination and an erythromycin resistance gene (*ermC*) for selection (Fig. 1). Such a five‑component fragment (UnprE, ermC, P_*mtlA*_, comk and DnprE) was prepared by two rounds of PCR. First, PCR with 2 × Phanta Flash Master Mix (Vazyme Biotech Co., Ltd.) was performed to prepare each fragment component. For preparation of the flanking fragments (UnprE and DnprE) of approximately 1000 bp each, P_mtlA_ and *comk,* the *B. subtilis* 6051a genome was used as the template, while the *ermC* fragment was obtained by PCR using the plasmid pMD19T‑aea as the template [26]. Next, all PCR products were treated with QuickCut™ DpnI (TaKaRa Biotechnology Co., Ltd.) and pooled together at the proper mole ratio (usually 1:1 between every two fragments) for overlapping PCR that generated the P_*mtlA*_‑comk cassette. Other linear DNAs, such as P_43_‑T7P, P_xylA_‑T7P, P_rpsF_‑T7P, P_T7_‑manC‑manB‑manA, or P_T7_‑gmd‑wcaG, were all prepared by similar strategies.

Recombination‑mediated genetic manipulations were performed by incubation of linear DNAs with corresponding* B*. *subtilis* competent cells, which was prepared as described by Ji et al., with the modification of 1% mannitol being used as the inducer instead of xylose [32]. Usually, 100 ng of plasmid or 1‑3 µg of linear DNA fragment was applied and incubated with 0.5 ml competence‑induced cell suspension for 120 min at 37 ℃. Then, 100 μL of regenerated cells were plated on solid medium and incubated overnight at 37 ℃. Colonies formed on selective plates were picked for colony PCR. The generated mutant strain was again transformed with plasmid pMK4‑cre to excise the selective marker *ermC* from the genome*.*

### WcaH modeling and docking

The WT WcaH model was built through Prime in Cite Schrodinger 2020‑3 (https://www.schrodinger.com/) using PDB structures 2I8T [22] and 2GT4 [39] as templates. Mg^2+^ and GDP‑mannose from the templates were kept in the WT WcaH model. Glide XP [40] was used in Cite Schrodinger 2020‑3 to simulate and dock GDP‑l-fucose and GDP‑mannose to the WT WcaH model. Modeling of WcaH mutants docked with GDP‑l-fucose was performed according to the conformation of WT WcaH bound with GDP‑l-fucose. Prime MM‑GBSA [41] (with the OPLS‑3e force field) was used to calculate the binding free energy for all docked complexes.

### Analytical and quantitative methods

The cell biomass was monitored by measuring the OD_600_ values of the cell suspension with an ultraviolet spectrophotometer, and the growth curve was fitted with a sigmoidal model. Detection of l-fucose and other related metabolites in the fermentation broth was conducted using a Shimadzu 20AVP HPLC system (Shimadzu Corp.) equipped with an RID‑10A refractive index detector. To prepare cell‑free samples, cultures were centrifuged at 12,000 ×*g* for 10 min and filtered using PTFE filters with an aperture span of 0.22 µm. The samples were then loaded into an Aminex HPX‑87H column (300 × 7.8 mm) (Bio‑Rad, USA) and separated by 5 mM H_2_SO_4_ solution as the mobile phase at a flow rate of 0.6 mL/min at 65 ℃. The L‑fucose concentration was calculated according to the calibrated standard curve. All data are representative of parallel independent experiments and presented as the means ± standard deviations (SDs). Statistical differences among the means of two or more groups (p < 0.05) were determined using one‑way analysis of variance.

## Supplementary Information

**Additional file 1: Figure S1.** *B*. *subtilis* growth experiment when supplied with **A **glucose **B** xylose **C **fucose **D** glycerol as the sole carbon source. **Figure S2. **L-Fucose standard** A **and fermentation supernatant **B** were analyzed by LC-MS. **Table S1.** The strains and plasmids used in this study. **Table S2.** The primers used in this study.

## Data Availability

All data generated or analyzed in this study are included in this published article. The datasets used and/or analyzed in the current study are available from the corresponding author upon reasonable request.
