# Supplementary material for: Rational design of GDP‑d‑mannose mannosyl hydrolase for microbial l‑fucose production
Source: Microb Cell Fact. 2023 Mar 24;22:56. doi: 10.1186/s12934-023-02060-y (PMC10037897; doi:10.1186/s12934-023-02060-y)
Supplement: Supplementary file 1 — Additional file 1: Figure S1. B. subtilis growth experiment when supplied with A glucose B xylose C fucose D glycerol as the sole carbon source. Figure S2. L-Fucose standard A and fermentation supernatant B were analyzed by LC-MS. Table S1. The strains and plasmids used in this study. Table S2. The primers used in this study. [file 12934_2023_2060_MOESM1_ESM.docx]

**Supplemental Information**


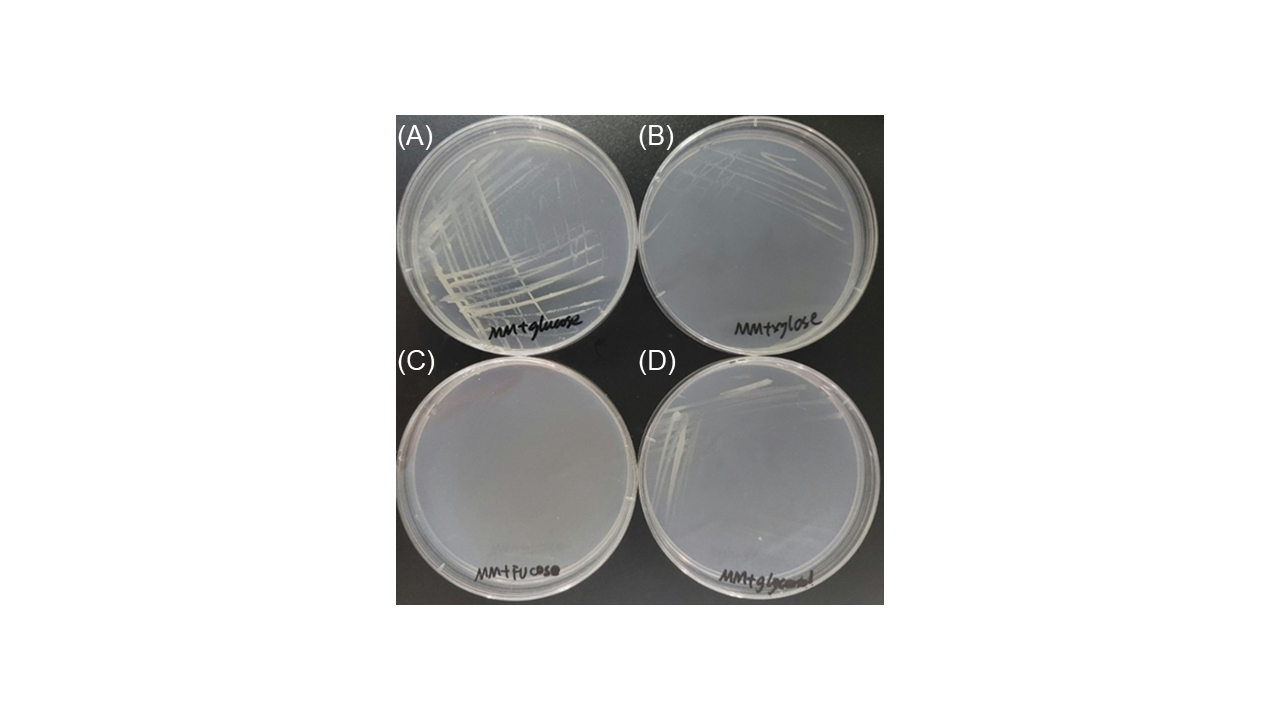


**Figure 1** *B*. *subtilis* growth experiment when supplied with **(A)** glucose **(B)** xylose **(C)** fucose **(D)** glycerol as the sole carbon source.


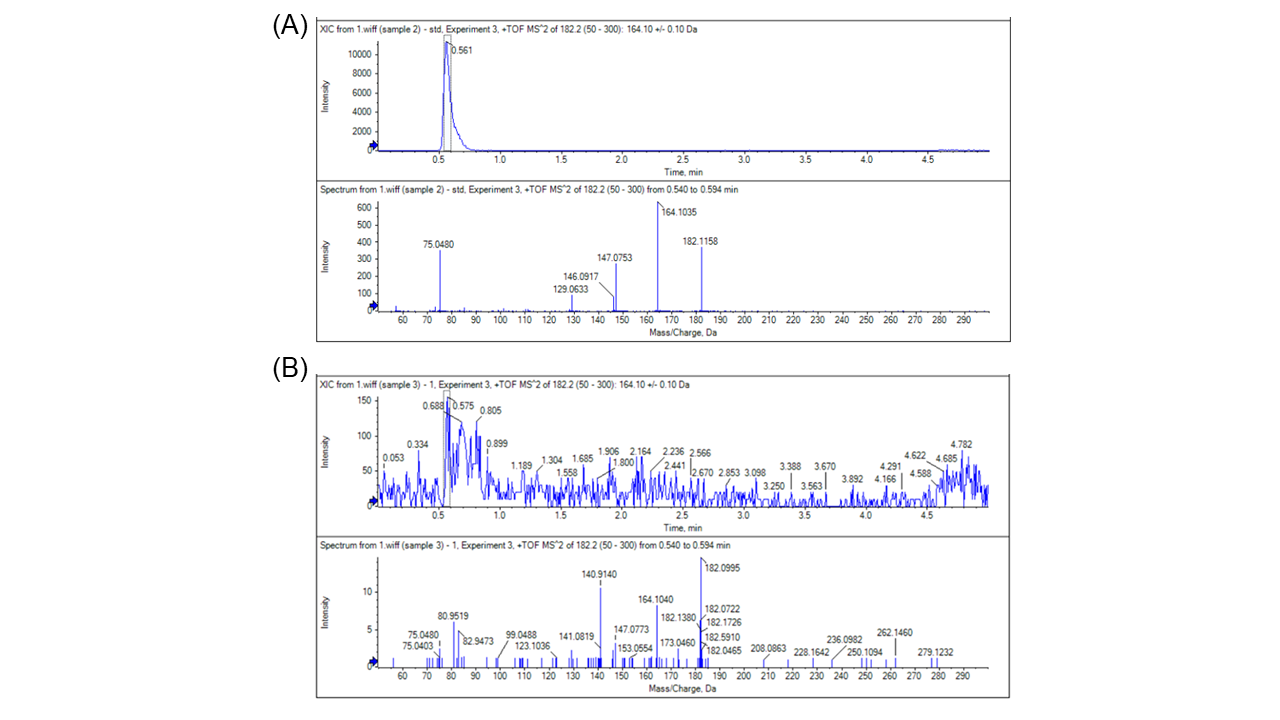


**Figure 2** L-Fucose standard **(A)** and fermentation supernatant **(B)** were analyzed by LC-MS.

**Table 1** The strains and plasmids used in this study

|  | Description | Source |
| --- | --- | --- |
| Strains |  |  |
| 164 | *B*. *subtilis* 6051a | Lab stock |
| 164M | 164 derivate, *nprE*::*P_mtlA_-comk*, | This study |
| 164MT | 164M derivate，*aprE*::*T7P* | This study |
| 164MCT | 164MC derivate, *aprE*:: *P_43_-T7P* | This study |
| 164MCX | 164MC derivate, *aprE*:: *P_xylA_-T7P* | This study |
| 164MCR | 164MC derivate, *aprE*:: *P_rpsF_-T7P* | This study |
| 164MTM | 164MT derivate, *manA*:: *P_T7_* *manC-manB-manA,* | This study |
| 164GF | 164MCTM derivate, *xylA*:: *P_T7_ gmd-wcaG* | This study |
| 164MCTGF | 164MC derivate, *aprE*:: *P_43_-T7P*, *manA*:: *P_T7_* *manC-manB-manA, xylA*:: *P_T7_ gmd-wcaG* | This study |
| 164MCXGF | 164MC derivate, *aprE*:: *P_xylA_-T7P, manA*:: *P_T7_* *manC-manB-manA, xylA*:: *P_T7_ gmd-wcaG* | This study |
| DH5α | *E*. *coli* for vector replication and preservation | Lab stock |
| Plasmid |  |  |
| pMK4-cre | Cm^R^, Shuttle vector pMK4 with *cre* under control of P*_spac_* | Lab stock |
| pMK4-comk | Cm^R^, Shuttle vector pMK4 with *comk* under control of P*_xylA_* | Lab stock |
| pMK4-T7 | Cm^R^, pMK4 carrying T7 promoter | This study |
| pMK4-T7gfp | Cm^R^, pMK4-T7 with *gfp* | This study |
| pMK4-T7wcaH | Cm^R^, pMK4-T7 with *wcaH* | This study |
| pMK4-R36Y | pMK4-T7 carrying wcaH^R36Y^ fragment | This study |
| pMK4-R36L | pMK4-T7 carrying wcaH^R36L^ fragment | This study |
| pMK4-R36F | pMK4-T7 carrying wcaH^R36F^ fragment | This study |
| pMK4-R36W | pMK4-T7 carrying wcaH^R36W^ fragment | This study |
| pMK4-R36I | pMK4-T7 carrying wcaH^R36I^ fragment | This study |
| pMK4-R36Y/N38R | pMK4-T7 carrying wcaH^R36Y/N38R^ fragment | This study |
| pMK4-R36Y/N38K | pMK4-T7 carrying wcaH^R36Y/N38K^ fragment | This study |
| pMK4-R36Y/L65R | pMK4-T7 carrying wcaH^R36Y/L65R^ fragment | This study |
| pMK4-R36Y/L65K | pMK4-T7 carrying wcaH^R36Y/L65K^ fragment | This study |
| pMK4-R36Y/F102R | pMK4-T7 carrying wcaH^R36Y/F102R^ fragment | This study |
| pMK4-R36Y/F102K | pMK4-T7 carrying wcaH^R36Y/F102K^ fragment | This study |
| pMK4-R36F/N38R | pMK4-T7 carrying wcaH^R36Y/N38R^ fragment | This study |
| pMK4-R36F/N38K | pMK4-T7 carrying wcaH^R36Y/N38K^ fragment | This study |
| pMK4-R36F/L65R | pMK4-T7 carrying wcaH^R36Y/L65R^ fragment | This study |
| pMK4-R36F/L65K | pMK4-T7 carrying wcaH^R36Y/L65K^ fragment | This study |
| pMK4-R36F/F102R | pMK4-T7 carrying wcaH^R36Y/F102R^ fragment | This study |
| pMK4-R36F/F102K | pMK4-T7 carrying wcaH^R36Y/F102K^ fragment | This study |
| pMD19T-aea | Amp^R^, pMD19T carrying *ermC* cassette | Lab stock |

**Table 2** The primers used in this study.

| Primers | Sequence |
| --- | --- |
| npr1F | gtgtttcgtccgcataatcaaaaacaatagagc |
| npr1R | taaaaataaaaaggctcctggtttattaggaaaagcctgagatccctcagg |
| PmtlAF | agggatctcaggcttttcctaataaaccaggagcctttttatttttaaaaaattgtcac |
| PmtlAR | aaggtgcgtctgttttctgactcatatataaaccctccctgttttgtttgtcgc |
| ComkF | caaacaaaacagggagggtttatatatgagtcagaaaacagacgcacctt |
| ComkR | cggtagcggccgcaagcttggatcccatatgactttggatccaagagaatat |
| comErmF | attctcttggatccaaagtcatatgggatccaagcttgcggccgctaccg |
| comErmR | cagtattttcaaaaagggggatttatttaagttagcccgggcatatgtaccgt |
| npr2F | gtacatatgcccgggctaacttaaataaatccccctttttgaaaatactg |
| npr2R | catgacagccatcgtcacccacttattc |
| apr1F | ACGACGGCCAGTGAATTCcatcgcttcttttaacgaaagattc |
| PaprER | ctttgatttttagatatctctttaccctctccttttaaaaaaa |
| T7PF | gatatctaaaaatcaaagggggaaatgg |
| T7PR | ttacgcgaacgcgaagtccgactct |
| T7ErmF | gacttcgcgttcgcgtaaggatccaagcttgcggccgctaccg |
| T7ErmR | taagttagcccgggcatatgtaccg |
| apr2F | tatgcccgggctaacttatagtaaaaagaagcaggttcctcca |
| apr2R | TCGACGGGCCCGGGATCCgccagctgggctaaggatcaggtta |
| P43-F | tgcttggcgaatgttcattcaaaagcttcgtgcatgcaggccg |
| P43-R | ctttgatttttagatatcgtgtacattcctctcttacctataa |
| PrpsF-F | tgcttggcgaatgttcatagttgcttatgaggatcttcttgcg |
| PrpsF-R | ctttgatttttagatatcctgtttgcacctccttttggactaa |
| Pxyl-F | catatctaatattataactaaattt |
| Pxyl-R | tttccccctttgatttaagt |
| UmanF | gcctgtagctcaaattttcgcctct |
| UmanR | gcggccgcaagcttaagcttaaaaagaaaatcccccgctttattcgattt |
| manErmF | aaatcgaataaagcgggggattttctttttaagcttaagcttgcggccgc |
| manErmR | aagatcgggctcgccacgaattcggtacccccgggcatatgtaccg |
| T7manBF | ttcgtggcgagcccgatcttccccatcggtgatg |
| manCR | cctccttactcgttcagcaacgtcagcaga |
| manAF | ggaggaactactatgacgactgaaccgttatttttca |
| manAR | gccctgccatgttacagatgggagacgataca |
| UxylF | ccgcaactgcatttaggaccattaa |
| UxylR | ttctttttaggatccctgcaggtgatttcccccttaaaaataaattc |
| xylErmF | atttttaagggggaaatcacctgcagggatcctaaaaagaagcag |
| xylErmR | tggggaagatcgggctcgccacgaattcggtacccccgggcatatg |
| T7gmdF | ccgaattcgtggcgagcccgatcttccccatc |
| wcaGR | tacccccgaaagcggtcttgattctca |
| DxylF | tgatgttattgtctggagatcaaccg |
| DxylR | tcaagatactcactctaatga |
| pMK4-F | gaattcctgctaacaaagcccgaaagg |
| pMK4-R | ggtatatcctcctttcttaaagtta |
| WcaHF | aagaaaggaggatataccatgtttttacgtcaggaagactttgc |
| WcaHR | ctttgttagcaggaattcttataatccgggtactccggtacgc |
| WcaH-R36Y-f | tcgcggcgagtttctgcttggcaaatacaccaaccgcccggcgcagggttact |
| WcaH-R36Y-r | agtaaccctgcgccgggcggttggtgtatttgccaagcagaaactcgccgcga |
| WcaH-R36F-f | agtcgcggcgagtttctgcttggcaaatttaccaaccgcccggcgcagggttactgg |
| WcaH-R36Y-r | taaccctgcgccgggcggttggtaaatttgccaagcagaaactcgc |
| Wcah-R36L-f | gagtttctgcttggcaaactgaccaaccgcccggcgcagggtt |
| Wcah-R36L-r | aaccctgcgccgggcggttggtcagtttgccaagcagaaactc |
| R36I-F | ggcaaaataaccaaccgcccggcgcagggttactg |
| R36I-R | gcggttggttattttgccaagcaga |
| R36W-F | ggcaaaTGGaccaaccgcccggcgcagggttactg |
| R36W-R | gcggttggtCCAtttgccaagcaga |
| N38R-f | acccggcgcccggcgcagggttactgg |
| N38R-r | ccagtaaccctgcgccgggcgccgggt |
| N38K-f | accaaacgcccggcgcagggttactggt |
| N38K-f | accagtaaccctgcgccgggcgtttggt |
| L65R-f | aagccgcatttgagcggcgcacgatggcggaactggggct |
| L65R-r | agccccagttccgccatcgtgcgccgctcaaatgcggctt |
| L65K-f | gccgcatttgagcggaaaacgatggcggaactggggctgcgt |
| L65K-r | acgcagccccagttccgccatcgttttccgctcaaatgcggc |
| Y102R-f | cactcacagagtggtgctcggttttcg |
| Y102R-r | cgaaaaccgagcaccactctgtgagtg |
| Y102K-f | accactcacaaagtggtgctcggttttcg |
| Y102K-r | cgaaaaccgagcaccactttgtgagtggt |
